# Editorial: Innate immunity and neurodegenerative diseases – triggers from self and non-self

**DOI:** 10.3389/fnmol.2023.1227896

**Published:** 2023-06-07

**Authors:** Jonathan Hulse, Adam Bachstetter, Surojit Paul, Kiran Bhaskar

**Affiliations:** ^1^Department of Molecular Genetics and Microbiology, University of New Mexico, Albuquerque, NM, United States; ^2^Department of Neuroscience, Sanders-Brown Center on Aging, University of Kentucky, Lexington, KY, United States; ^3^Department of Neurology, University of New Mexico, Albuquerque, NM, United States

**Keywords:** neurodegenerative disease, innate immunity, neuroinflammation, neurological disease, blood-brain barrier disruption

While innate immune responses are typically short-lived, resolving upon clearance of the inciting pathogen or damage-associated molecular pattern, unresolved innate immune responses in the central nervous system (CNS) promote toxic inflammatory conditions that mediate neurodegenerative diseases. Understanding the cellular mechanisms that mediate the transition from neuroprotective immune responses to toxic chronic inflammatory responses in the CNS is a major area of ongoing research. Furthermore, neuroinflammatory signaling in neurological disease is a self-propagating process, worsening the disease state over time as additional immune cells are recruited and polarized toward inflammatory phenotypes, blood-brain barrier (BBB) function is disrupted, and neurodegeneration progresses. This Research Topic includes 14 peer-reviewed articles published in different Frontiers journals. Five are comprehensive reviews on the role of inflammatory alterations in neurological and neuropsychiatric diseases. The remaining nine articles are original research on innate immune activation in spinal cord injury, ischemia, and other neurological and neuroinflammatory conditions. This editorial summarizes these published reports and how they may advance our knowledge of innate immune activation driven by self- and non-self-triggers (Figure 1). Furthermore, we also attempt to identify gaps in knowledge so that future research can focus on these unexplored areas.

**Figure 1:**
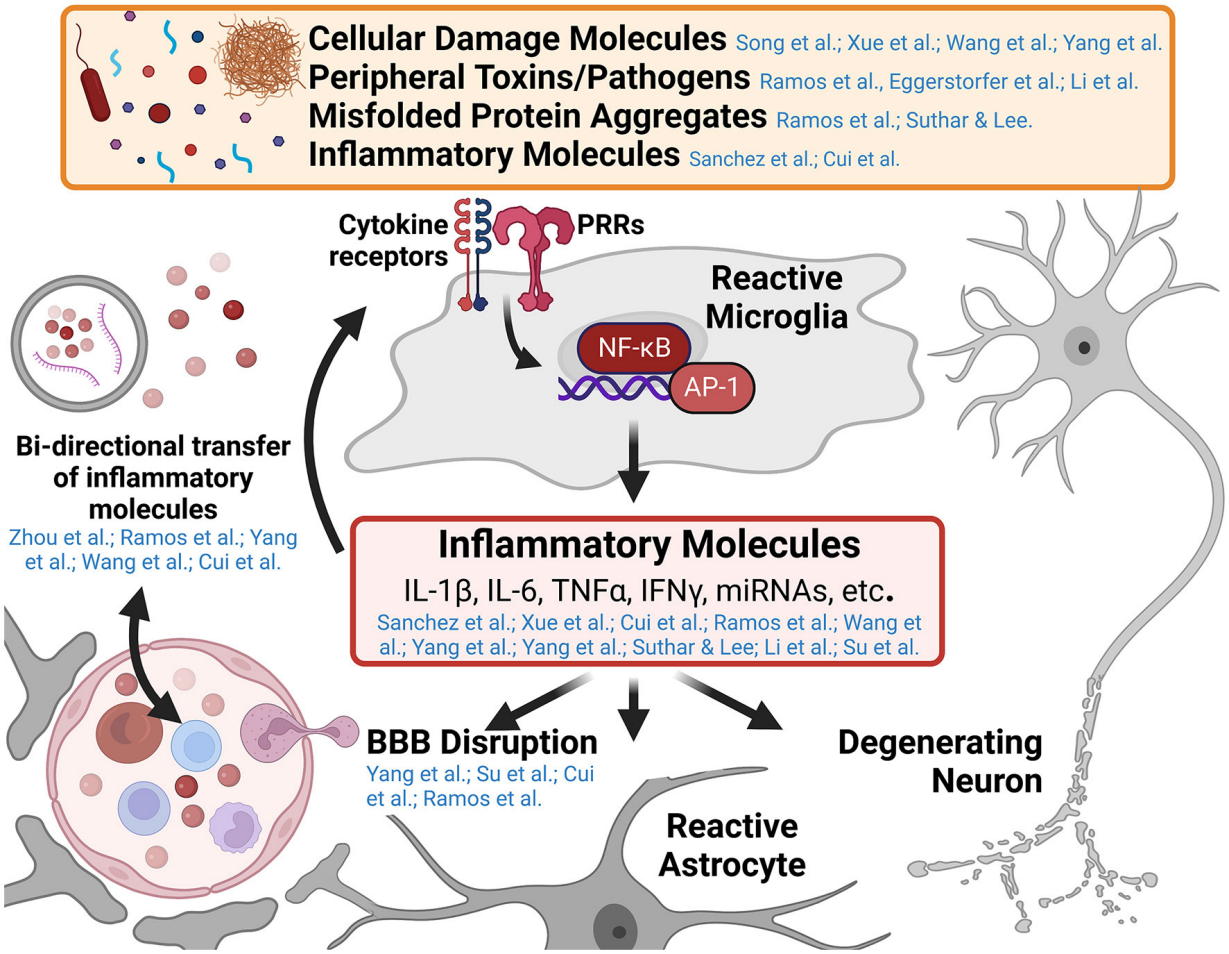
Graphical abstract of major concepts and themes explored in this Research Topic. Microglia are the primary innate immune cell of the central nervous system (CNS) and respond to numerous pathogen and damage associated molecular patterns in various neurological diseases. Pattern recognition receptors (PRRs) such as Toll-like receptors and Nod-like receptors, recognize the presence of a variety of inflammatory molecules such as cellular damage debris, peripheral toxins and pathogens (e.g., alcohol and bacterial lipopolysaccharide), and misfolded protein aggregates (e.g., amyloid-β, tau, and α-synuclein aggregates). These trigger signaling cascades resulting in the activation of inflammatory genes by transcription factors like nuclear factor kappa-B (NF-κB) and activating protein-1 (AP-1). The release of inflammatory molecules from reactive microglia effects numerous cell types within the CNS including enhancing inflammatory microglial responses, altering astrocyte function, promoting blood-brain barrier disruption and recruiting peripheral immune invasion, and promoting the degeneration and dysfunction of neurons (a central theme of this Research Topic). Several papers in this collection highlight the cross-talk between the innate immune system of the CNS and the peripheral immune system in the development of neuroinflammatory and neurodegenerative diseases. This figure was created using BioRender.com.

To start with, the nature of innate immune responses and the production of inflammatory cytokines is to recruit a more robust immune response from additional cells. This is highlighted by Sánchez et al. in their study on the effects of the innate immune molecules apoptosis-associated speck-like protein containing a CARD (ASC) and matrix metalloprotease 10 (MMP10) on inducing an inflammatory microglial phenotype and further upregulating inflammatory cytokine signaling. This study, and many others in this Research Topic demonstrate how innate inflammatory signaling molecules in the CNS contribute to pathological feed-forward processes in neurodegenerative diseases and identify many nodes for future therapeutic targeting.

Tissue injury in the CNS, such as mechanical trauma, environmental toxins, hypoxic insult, or hemorrhage, is a major instigator of innate immune processes, including toxic neuroinflammation, that worsens neurodegeneration. The role of innate immunity in response to each of these modes of CNS injury is explored in this Research Topic. Xue et al. demonstrate the role of innate immune processes in mechanical spinal cord injury for mediating allodynia, highlighting the role of the RIPK-NF-κB-NLRP3 pathway for triggering inflammatory signaling in response to cellular damage signals even at sites away from the primary site of mechanical tissue injury. Ramos et al. review the mechanism of ethanol toxicity-induced neurodegeneration, highlighting the role of ethanol reactive microglia (ERMs) and innate immune neuroinflammatory processes as a key mediators of ethanol-induced neurotoxicity with parallels to Alzheimer's disease (AD) and potential contributions to AD etiology. Wang et al. identify a novel cytokine, Tissue Non-specific Alkaline Phosphatase, that plays a substantial role in the NF-κB-mediated inflammatory processes in response to the hypoxic injury related to the development of Spastic Cerebral Palsy. Finally, Yang G. et al. provide a detailed review of the innate immune signaling mechanisms in microglia in response to intracerebral hemorrhage that confers protection, promote resolution and cleanup of toxic blood products, or drive neuroinflammation that worsens neurodegenerative processes after a hemorrhagic stroke.

The BBB is the primary interface between the CNS and the peripheral immune system. The neurovascular unit, which forms the BBB, comprises several cell types, including a monolayer of tightly sealed vascular endothelial cells with a basement membrane, surrounding pericytes, and a covering layer of astrocyte end feet. This selectively permeable barrier protects the CNS from circulating toxins, pathogens, and damaging immune cells and inflammatory mediators and was believed to maintain the CNS as an immune-privileged site. More recently, researchers have appreciated the importance of a coordinated cross-talk between the peripheral immune system and the CNS in health and disease.

In this Research Topic, Su et al. assert that markers of increased BBB permeability and peripheral immune cell recruitment are signatures of normal aging across brain regions coinciding with increased molecular signatures of neuroinflammatory signaling. Disruption of the BBB with increased inflammatory infiltrate is a common pathological phenomenon in numerous disease states related to neurodegeneration. Interestingly, BBB disruption in response to inflammatory signaling is highlighted in Multiple Sclerosis (MS; Cui et al.), alcohol use disorder (AUD), and AD (Ramos et al.). In each of these disease states, the inflammatory cytokine interleukin-1β (IL-1β) is a key culprit in BBB dysfunction along with interleukin-6 (IL-6) and tumor necrosis factor-α (TNFα). These three critical inflammatory cytokine mediators of BBB disruption are explored in detail by Yang J. et al., highlighting the known molecular signaling mechanisms by which BBB integrity is disrupted, peripheral immune cells are recruited to the CNS, and inflammatory signaling in microglia and peripheral immune cells are upregulated.

Cross-talk between the peripheral immune system and the innate immune system of the CNS involving the transfer of inflammatory signaling molecules across the BBB is described throughout this Research Topic. Zhou et al. describe brain-derived small extracellular vesicles (sEVs) circulating in the plasma after acute cerebral ischemia containing brain-specific micro-ribonucleic acids (miRNAs). Temporal dynamics of these sEVs indicated that their presence in the peripheral circulation correlated with BBB disruption and could serve as a marker of disease staging and progression. Conversely, Ramos et al. describe the potential role of peripherally derived sEVs containing inflammatory cargo from liver in AUD that can modulate CNS neuroinflammatory processes. In many disease states, peripheral inflammation may accompany and contribute to neuroinflammation and vice versa. Increases in peripheral inflammatory molecules and immune cells in response to neuroinflammatory processes in the brain are discussed in the context of MS (Cui et al.), AUD and AD (Ramos et al.), Spastic Cerebral Palsy (Wang et al.), and intracerebral hemorrhage (Yang G. et al.). Eggerstorfer et al. and Li et al. both assert that peripheral inflammation, such as a peripheral bacterial lipopolysaccharide endotoxin (LPS) exposure in mice or during illness in humans, contributes to neuroinflammatory processes in the CNS that contribute to neuronal dysfunction.

Major Depressive Disorder (MDD) is the topic of two articles in this Research Topic (Eggerstorfer et al.; Li et al.). In a meta-analysis of eight separate imaging studies, Eggerstorfer et al. demonstrate that Translocator protein (TSPO), a marker of neuroinflammation, is elevated in several brain regions associated with disease symptoms in human patients assessed using positron emission tomography (PET). Li et al. dive into the mechanism of hypoxia-inducible factor 1 (HIF-1) and phosphoinositide 3 kinase (PI3K) signaling in response to LPS-induced peripheral inflammation for regulating depressive behaviors in rodent models. This study also demonstrated that repurposing of the FDA-approved HIF-1 stabilizing agent, FG-4592, could reduce depressive symptoms, neuroinflammation, and synaptic defects in rodents stimulated with LPS. These studies further support a new understanding of the role of neuroinflammation in mediating MDD, establishing a potential therapeutic target outside of monoamine neurotransmitter modulation that may yield improvements for many patients with MDD that are refractory to current pharmaceutical therapies.

While this editorial is not aimed to cover all 14 articles in this Research Topic, with some articles slightly outside the theme yet highly relevant to neuronal homeostasis, we express our gratitude to the many authors, reviewers, and editors around the world that contributed to this Research Topic to further our understanding of innate immunity in neurodegenerative diseases. We hope that readers will enjoy reading it.

## Author contributions

JH reviewed the articles published on this Research Topic, drafted the manuscript, and created the figure. AB, SP, and KB read through published articles and edited the manuscript and figures. All authors contributed to the article and approved the submitted version.

